# Accountable Artificial Intelligence: Holding Algorithms to Account

**DOI:** 10.1111/puar.13293

**Published:** 2020-11-11

**Authors:** Madalina Busuioc

**Affiliations:** ^1^ Leiden University

## Abstract

Artificial intelligence (AI) algorithms govern in subtle yet fundamental ways the way we live and are transforming our societies. The promise of efficient, low‐cost, or “neutral” solutions harnessing the potential of big data has led public bodies to adopt algorithmic systems in the provision of public services. As AI algorithms have permeated high‐stakes aspects of our public existence—from hiring and education decisions to the governmental use of enforcement powers (policing) or liberty‐restricting decisions (bail and sentencing)—this necessarily raises important accountability questions: What accountability challenges do AI algorithmic systems bring with them, and how can we safeguard accountability in algorithmic decision‐making? Drawing on a decidedly public administration perspective, and given the current challenges that have thus far become manifest in the field, we critically reflect on and map out in a conceptually guided manner the implications of these systems, and the limitations they pose, for public accountability.

**Evidence for Practice**

- The article provides public sector practitioners with insight into the distinct accountability challenges associated with the use of AI systems in public sector decision‐making.
- It digests and explicitly links technical discussions on black‐box algorithms as well as explainable AI and interpretable models—different approaches aimed at model understandability—to public accountability considerations relevant for public bodies.
- It provides specific policy recommendations to securing algorithmic accountability—prominent among these, the importance of giving preference to transparent, interpretable models in the public sector over black‐box alternatives (whether in a proprietary or in a technical sense, i.e., deep learning models).
- This will become critical to administrators’ ability to maintain oversight of system functioning as well as to their ability to discharge their account‐giving duties to citizens for algorithmic decision‐making.

A proprietary algorithm widely used by US courts to predict recidivism in both bail  and sentencing decisions was flagged by *ProPublica* as biased against black defendants (Angwin et al. 2016); natural language processing (NLP) algorithms for textual analysis can display recurrent gender biases (Bolukbasi et al. 2016), for instance, associating the word “doctor” with “father” and “nurse” with “mother”; facial recognition algorithms have persistently been found to display much higher error rates for minorities (Buolamwini and Gebru 2018; Lohr 2018; Snow 2018; Medium 2019), potentially leading to false arrests and discrimination of already marginalized groups when used in policing (e.g., Garvie and Frankle 2016); algorithms used for university admissions to predict exam grades have recently shown serious failures, with disparate negative effects on high‐achieving students from disadvantaged backgrounds (Broussard 2020; Katwala 2020). These are only a few of the growing number of examples of bias encountered in algorithmic systems used not only in private but also public sectors.

While simultaneously algorithmic systems based on artificial intelligence (AI) are undoubtedly associated with tremendous technological innovation, and are foreshadowed “to supercharge the process of discovery” (Appenzeller 2017), the examples above underscore the importance of oversight of AI algorithmic decision‐making. As algorithmic systems have become increasingly ubiquitous in the public sector, they raise important concerns about meaningful oversight and accountability (Bullock 2019; Diakopoulos 2014; European Parliament Study 2019; Pasquale 2015; Yeung 2018; Yeung and Lodge 2019; Young, Bullock, and Lecy 2019) and the need to identify and diagnose where the potential for accountability deficits associated with these systems might—first and foremost—lie.

This is precisely the topic this paper seeks to address. We start off with a brief discussion of the rise of AI algorithms and a key subset thereof, machine learning (ML) algorithms, and their prevalence in informing (and transforming) public sector decision‐making, followed by a conceptual discussion on accountability. We next apply this conceptual framework to systemically diagnose and analyze the locus of the distinct accountability challenges associated with AI use in public sector decision‐making as well as to provide initial clues to possible mitigating steps forward. We structure the analysis along the three phases that span an accountability process: information, explanation, and (the possibility for) consequences (Bovens 2007). The paper adopts a public administration perspective on the topic, but draws on broader multi‐disciplinary literature, including relevant computer science literature, on the state of play on core aspects crucial to account‐holding (specifically, explanation and interpretability of AI algorithms) so as to adequately ground our discussion within the technical realities and challenges at play in securing accountability.

## The Rise of Algorithms and the Need for Countervailing Checks

While not manifest in the doomsday apocalyptic scenarios that have received much media coverage, AI is here. Artificial intelligence algorithms govern in more subtle yet fundamental ways the way we live and are transforming our societies. Tremendous technological advances brought on by data, computation, and the growing power of machine pattern recognition—relying on a range of methods referred to as “deep learning” or “neural networks”—have led to the ubiquity of artificial intelligence algorithms in structuring technological but also human interactions.

This technology is present in an array of mundane applications: from text predictors on our mobile phones, or speech‐to‐speech translations, to the algorithms that recommend films to us on Netflix. But it is not restricted to these commonplace, “low‐stakes” applications. Algorithms also govern the operation of our internet search engines—they retrieve and prioritize search information and ultimately, therefore, what information is available to users—and of social media. High‐frequency trading algorithms run our electronic markets; algorithms inform automated individual credit decisions and key health decisions, and govern semi‐autonomous driving (Pasquale 2015). They are also anticipated to next encode ethical life‐and‐death decisions (e.g., in case of a crash, should a self‐driving car prioritize saving its driver/passengers or pedestrians; Bonnefon, Shariff, and Rahwan 2016).

Importantly, such algorithms are increasingly not only the purview of private actors—that drive innovation in this area—but are also widely relied upon by governments and public (regulatory) bodies (AI Now Institute NYU 2018; Citron 2008; Eubanks 2018; European Parliament Study 2019; O'Neil 2016; Richardson, Schultz, and Crawford 2019; Shark 2019; UK Government Office for Science 2016; Yeung 2018; Yeung and Lodge 2019). The promise of efficient, low‐cost, or “neutral” solutions harnessing the potential of big data has led public bodies to adopt algorithmic systems in the provision of public services (Eubanks 2018; Ferguson 2017; O'Neil 2016), with fundamental implications for public administration: The impact of digital technologies on the public sector has been well recognized as transformative (Bovens and Zouridis 2002), and AI is set to significantly accelerate and deepen such processes (Shark and Shropshire 2019; Young, Bullock, and Lecy 2019), fundamentally impacting not only how public services are structured but importantly also our public service values, the exercise of administrative discretion and professional judgment (Barth and Arnold 1999; Bullock 2019; Busch and Henriksen 2018; Young, Bullock, and Lecy 2019), and in doing so, the very nature of public bureaucracies.

Telling examples of high‐stakes applications in public sector decision‐making already abound: Algorithmic tools are increasingly relied upon by police departments to predict crime and inform policing decisions (Ferguson 2017). Such systems are especially common in the United States, but similar predictive techniques are also deployed by police forces in other jurisdictions, such as the Crime Anticipation System (CAS) used by the Dutch police or a variety of predictive policing technologies in use, or anticipated to be used, by at least 14 police forces in the UK (Kelion 2019). Algorithms have also been deployed by school districts for teacher appraisals and to inform school reforms and individual firing decisions (O'Neil 2016), by fire departments to predict where fires will break out and to prioritize inspections (Diakopoulos 2014), in criminal justice (Angwin et al. 2016), and in a whole array of other areas as varied as healthcare (e.g., NHS collaborations with both DeepMind and recently, Palantir), immigration, or public benefits (AI Now Institute NYU 2018; Citron 2008; Eubanks 2018).

Their use has not been deprived of controversy. Widely documented high‐stakes failures abound: “there have been cases of people incorrectly denied parole, poor bail decisions leading to the release of dangerous criminals, ML‐based pollution models stating that highly polluted air was safe to breathe and generally poor use of limited valuable resources in criminal justice, medicine, energy reliability, finance and in other domains” (Rudin 2019). Especially disconcerting, AI algorithms have been revealed to reproduce historical biases hidden within their training data. For instance, a recent study in the US context revealed that predictive systems used by police departments in several jurisdictions are likely built on discriminatory and unlawful historical police practices (Richardson, Schultz, and Crawford 2019). “Dirty data” (due to systematic police underreporting or logging of specific types of crimes, systemic over‐policing of certain areas, or minorities), encoded in historical datasets, will necessarily “corrupt” and similarly bias the predictive systems relying on these data points as training data (ibid).

Relatedly, AI algorithms have also been found to get caught in negative feedback loops that are difficult to spot, break out of, and/or self‐correct. For instance, if an algorithm were to wrongly predict a particular area as “high crime,” the resulting enhanced police presence will result in more arrests in that area, which become the algorithm's new training data, reconfirming and reinforcing its earlier predictions. This becomes especially problematic if the initial predictions are biased, e.g., due to human bias in the training data (e.g., a historically overpoliced neighborhood): the algorithms' predictions become “self‐fulfilling prophecies.” At the same time, algorithmic biases and misfunctioning have proven difficult to diagnose and challenge due to the reputed black‐box operation of such systems as well as inherent complexity.

This risks rendering the “cure” worse than the “disease”: Historical discrimination and human biases get propagated by automation while simultaneously becoming harder to spot and challenge, the same biases now repackaged in algorithmic black‐boxes with a seeming veneer of neutrality and mathematical objectivity. As a result, calls for mechanisms to improve AI algorithm transparency and for public regulation are sharply on the rise—for instance, of facial recognition (e.g., by leading computer scientists; see the open letter signed by 78 AI researchers, Medium 2019) or even calls for a moratorium on the technology's use (e.g., UK House of Commons Science and Technology Committee 2019), as well as recent industry‐led moratoriums on its use in policing (Amazon, Mircrosoft) and market exits (IBM).

This is not meant, and should not be read, as an indictment of AI algorithms and their use. At their best, algorithms stand to usher in tremendous technological potential and efficiency gains. Moreover, much algorithm use will inform routine “low‐stakes” operations whose functioning (or misfunctioning) will not raise much cause for concern. At the same time, however, it is important to acknowledge that AI algorithms stand to have—and are having—very real and ground‐breaking societal and individual‐level implications. It is the rise of “algorithmic power” (Diakopoulos 2014), the fact that “authority is increasingly expressed algorithmically” (Pasquale 2015, 8), that brings with it the corresponding need for oversight of such power. As AI algorithms have permeated key public aspects of our existence, this necessarily raises important accountability questions: What accountability challenges do AI algorithmic systems bring with them, what specific deficits arise through their operation, and how can we safeguard accountability in algorithmic decision‐making in the public sector?

Drawing on a decidedly public administration perspective, and given the challenges that have thus far become manifest in the field, we reflect critically, and in a conceptually guided manner, on the implications of these systems for public accountability. Such a perspective is direly needed: while algorithms increasingly help mediate the relationship between individuals and public bodies in fundamental ways, and while accountability is becoming a topic of central concern to algorithmic discussions and a key concern for practice, for the most part, these emerging debates have lacked much‐needed input from our field.

## Accountability: A Conceptual Frame

“Accountability refers to being answerable to somebody else, being obligated to explain and justify action and inaction” (Olsen 2014, 107). The exercise of power—the ability to yield power—is said to demand accountability. Yet, what accountability might entail is not always straightforward, even in more traditional contexts. Precisely for this reason, in recent years, there have been growing efforts by public administration scholars to arrive at, and converge upon, a “narrow” or “minimal definition” of public accountability to facilitate its study in practice (Bovens 2007; Bovens, Schillemans, and Goodin 2014). In this understanding, accountability is defined as “a relationship between an actor and a forum, in which the actor has the obligation to explain and justify his or her conduct, the forum can pose questions and pass judgement, and the actor might face consequences” (Bovens 2007, 450). While elaborated with more traditional accountability contexts in mind (such as political–bureaucratic relations), this conceptualization appears directly applicable and pertinent to algorithmic decision‐making.

Essentially, a meaningful accountability process is composed, we are told, of three phases: *information*, *explanation or justification*, and (the possibility for) *consequences*. These “requirements for effective accountability” (Olsen 2014, 113) are exercised towards an external authority. In other words, there must be an obligation, whether formal or informal, on the part of the *actor—*be it a bureaucratic agent, a private provider, a public sector body—to render account to the *accountability forum*. Accountability is relational (Bovens 2007; Romzek and Dubnick 1987; Schillemans and Busuioc 2015), and a variety of relationships can be conceptualized in this manner: for instance, in a bureaucratic context, an agency gives account to a variety of forums ranging from its parent ministry, the parliament, citizens to courts or the media etc. In the context of AI, such forums could conceivably similarly include traditional institutional forums such as courts, parliamentary committees, ombudsmen, etc. but also purpose‐built forums such as AI ethics, standardization, and audit bodies, monitoring AI system design and operation.

Within extant understandings, to operate effectively any accountability process needs first and foremost *information—*essentially, transparency of agent conduct or performance (Bovens 2007). This serves the purpose of mitigating “informational asymmetries,” affording insight into actor decisions and actions (or inactions). While necessary, in and of itself transparency is *not* a sufficient condition for accountability (Bovens 2007). Accountability is closely linked to “answerability,” and a key element thereof is that of *explanation* or justification. In a political context for instance, parliamentary committees can hold hearings with organizational actors under their purview to ask questions and seek explanations for observed failings. Similarly, public organizations make public statements or issue reports to justify specific actions to the broader public.

Information and explanation requirements serve as the foundation that allows the accountability forum to assess and judge whether decisions or actions undertaken were appropriate. For meaningful accountability to have taken place—and to the extent that failings have been identified—there should be a possibility to extract *consequences*. Negative judgment can result in the imposition of sanctions and the need for actors to undertake remedial actions (or rectification) to address failures and afford redress to those adversely affected (Bovens 2007; Mulgan 2000).

This is not to say, however, that accountability criteria and standards are always clear‐cut. The practice of public accountability involves complex trade‐offs among contending goals, claims, and normative criteria as well as among external expectations (Busuioc and Lodge 2017; Romzek and Dubnick 1987). The standards will be particularly fluid in “unchartered waters” such as unsettled polities or other *non‐routine situations* such as around new technologies (Olsen 2014). Unlike settled arenas where “attribution of accountability is guided by social‐validated (…) rules, routines, procedures, doctrines, expectations” (Olsen 2014, 111), in unchartered areas “actors have to learn their place in the larger political order through experience with what is seen as acceptable and politically possible” (Olsen 2014, 115). This is also precisely why interrogation and explanation are so crucial to accountability. Arriving at an enhanced understanding of “what is seen as acceptable” requires making tensions and trade‐offs explicit and subject to interrogation, debate, and justification (Olsen 2014, 115).

We rely on the Bovens (2007) conceptualization above as a starting point to anchor our analysis of accountability of AI algorithmic decision‐making in the public sector and emerging challenges. This allows us to pin down the concept to a set of identifiable characteristics and to broadly map out and locate the sources of emerging deficits at different phases of the accountability process. Our focus is systemic—we do not aim to zoom in on specific micro‐level (actor–forum) relationships, but rather set out to capture a bird's‐eye view of the challenges that arise along the three phases. Particularly given the discussion above on non‐routine contexts, we take a first stab at diagnosing the systemic challenges that (are set to) arise to the effective functioning of accountability systems when applied in this area. We delve into these matters next, but not before first settling, for the sake of the clarity of our argument, a few definitional aspects.

## AI, Algorithmic Decision‐Making, and Power: Mind the Emerging Accountability Gaps

An *algorithm* is essentially any set of rules (whether computational or other) implemented in sequence to reach a particular outcome. What sets apart the algorithms that are at the heart of our investigation is that such algorithms learn the rules that govern their behavior on their own. They discover hidden patterns in the data, pairing specific data inputs with outputs, effectively learning input–output relationships or “mappings,” by being unleashed on large amounts of training data (the so‐called training set). They use this training data to determine and modify the model—the various “features” contributing to an outcome and their corresponding weights (coefficients) which they then use to make predictions on as‐yet‐unseen data, using the learned input–output mappings. What renders them so pervasive (and popular) is that a whole range of problems can be framed in these input–output terms: from image recognition and its myriad of applications (e.g., video surveillance, DNA sequencing, or tumor mapping) to risk assessment models (on credit or crime) or recommender systems (for news, purchases, and social media).

Such algorithms are increasingly relied upon in public and private decision‐making. *Algorithmic decision‐making* refers to the use of algorithms as an aid or as a substitute to human analysis, to make or inform (and improve the quality of) decisions or actions. Algorithmic decision‐making can in principle be either *fully automated*, or it can be *mixed*, i.e., entail a human decision‐maker or reviewer in‐the‐loop (Citron 2008). While both types can occur in the public sector (Citron 2008), most commonly algorithms in the public sector tend to inform human decision‐making rather than make final fully automated decisions without a human intervener (see also Edwards and Veale 2017, 45). And in fact in some jurisdictions, such as the EU, there is a right (albeit subject to exceptions) to *not* be subject to a decision based solely on automated decision‐making (i.e., the EU General Data Protection Regulation, GDPR).

It is for this reason—empirical relevance—that our investigation focuses explicitly on *mixed decision‐making* where AI algorithms *inform* human decision‐making in the public sector. This is currently the relevant and pressing context of AI algorithm use in the public sector, especially so from an accountability perspective given the reliance thereon in non‐routine high‐stakes scenarios. When we speak of *actors* in an accountability sense therefore, we still in this context, as in traditional contexts, speak of human actors. As algorithms have not achieved sentience or consciousness—despite widely hyped media stories, there has been little discernible progress towards so‐called “artificial general intelligence” (AGI) or “human‐level AI” (Ng 2018)—the responsibility for algorithmic use and operation in the public sector necessarily lies with human actors: AI system providers and public sector adopters and users for the operation and the implications of the algorithmic systems they create and respectively purchase and deploy.

When we speak of mixed algorithmic decision‐making then, it is important to note that we necessarily speak of two levels: the AI algorithmic output, recommendation, or decision (and the algorithmic processes through which these were arrived at) *and* the subsequent interaction between the algorithmic output and the human decision‐maker. Algorithmic accountability for mixed systems thus pertains and requires the examination of two interrelated stages: the initial algorithmic results and how these were reached—i.e., transparency and justification of the AI model design, setup, and operation, which necessarily determine algorithmic results—*as well as* the role that these algorithmic recommendations play in human decisions and/or actions.

The latter aspect too, is particularly critical to meaningful oversight in this context and should not be underestimated. As we will see below, there is potential for unexpected new sources of bias that arise at the interface of the two. In other words, for decisions and actions undertaken on the basis of algorithmic inputs and assessments (e.g., decisions to direct police resources to particular areas on the basis of algorithm‐generated “heat maps”) to be open to meaningful interrogation, explanation, and justification requires unpacking *both* algorithmic processes *and* the human–algorithm interaction.

Below, we discuss, in turn, the systemic challenges that arise in this respect, structuring the discussion along the three requirements of accountability.

### Information: Asymmetries Compounded

With information being the “lifeblood of accountability,” oversight of ML algorithmic systems becomes especially challenging first and foremost, due to the tremendous information and expertise asymmetries that characterize them.

#### The Inherent Opaqueness of Deep Learning Tools

A particularly problematic feature—from an informational perspective—is the inherent opaqueness of many of these systems. Given particular inputs, we know the final algorithmic output, i.e. the ”classification” made (e.g., “high risk” or “low risk”), the decision reached (e.g., “credit denied”), or the specific outcome predicted (e.g., a risk assessment score). Yet, how the algorithm arrived at the particular predictive outcome—which subparts of the input data it finds important to predict an outcome—is often not transparent. Of course, not all AI algorithmic models need be opaque. While “black‐box” algorithms raise complex opaqueness and interpretability challenges, simpler interpretable ML algorithms do not, but have generally also been regarded as less powerful (Guidotti et al. 2019; see however, Rudin 2019).

Opaqueness is especially characteristic of “deep learning” algorithms, a key subset of ML algorithms, and the “primary driver of the revolution in AI” (Ford 2018, 10). Neural networks consist of multiple hidden (or intermediary) layers of artificial neurons that relate inputs to outputs, and are often referred to as “black‐boxes,” i.e., “systems that hide their internal logic to the user” (Guidotti et al. 2019). This system opaqueness is also due to the fact that, as the relevant “features” of the model (the parameters that contribute to a prediction) are identified by the system itself by sieving through large amounts of data, they can escape human interpretability—also that of its designers. The key to its effectiveness is precisely the source of its opaqueness: the features parsed by the machine might not be identifiable as valid or recognizable features to the human mind.

To illustrate with a simple example, the way a “classifier” (a deep learning classification algorithm) learns to classify a picture (e.g., as “cat” or “non‐cat”, a classic deep learning example) is through its different neurons learning different elements of a picture: while the first layer of neurons might learn to recognize diagonal lines, edges, or simple gradients, the next layer will take these inputs and combine them together into new inputs for the following layer, which will learn to recognize the presence of faces, etc. (Zeiler and Fergus 2014). Thus, the internal functioning of the algorithm (i.e., the functioning of its intermediate layers) is not intuitively scrutable to us, especially as neural networks go deeper (i.e., increase in number and architectural complexity). In fact, when the internal operation of the algorithm is reconstructed by computer scientists for visualization (see Zeiler and Fergus 2014), the visualized patterns of what the different layers of neurons actually do are not corresponding images but deconstructed superimposed layers thereof. In other words, when system operation is rendered visible, the features used by the model are not necessarily directly understandable or intuitive.

Depending on the respective algorithm, such a neural network can be hundreds of layers deep and entail thousands of features and millions of weights contributing to a predictive outcome. As a result, it is no longer identifiable which parts of the initial input data the algorithm finds important to predict a final outcome. Moreover, the data pertinent to the prediction outcome are no longer constituted by “just” the initial input variables but by thousands of sub‐features thereof, their weights, and their interactions.

Such algorithms are therefore, by virtue of their technical make‐up, highly non‐transparent—including to system engineers. Even assuming that the algorithm was (publicly) available, which as we will see below is a separate challenge (proprietary black‐boxes), this information might well not be intelligible (technical black‐boxes). This inbuilt opacity constitutes a distinctive—and especially “wicked”—informational problem pertaining to AI systems that differentiates and sets them apart from traditional forms of expertise, and one which is especially important to highlight when discussing oversight challenges associated with their use in public sector decision‐making.

#### Secrecy and Proprietary Algorithms

To make matters yet more difficult, information availability can be further limited by the fact that algorithms are often proprietary, including when used in the public sector. Developed and sold by private for‐profit companies, the workings of commercial algorithms are often not publicly divulged (Carlson 2017; Citron 2008; Pasquale 2011). Pasquale (2011, 237) traces a shift in this context from “legitimacy‐via‐transparency to reassurance‐via‐secrecy” with “profoundly troubling implications for the foundations of social order in the information age.” And reiterated: “the law is presently stacking the deck against accountability for automated authorities” (ibid).

Thus, even if the algorithm's features and operations are understandable such as with simple AI algorithms like decision trees, these can still be secretive for proprietary reasons: “Trade secrets are a core impediment to understanding automated authority like algorithms” (Diakopoulos 2014, 12). Trade secrets exemptions limit information access rights both in the US Freedom of Information Act (FOIA) (Diakopoulos 2014) as well as in the EU GDPR (Wachter, Mittelstadt, and Floridi 2017). The FOIA trade secrets exemption, for instance, “allows the federal government to deny requests for transparency concerning any third party software integrated into its systems” (Diakopoulos 2014, 12), and trade secret protections are being put forward by algorithmic makers to escape disclosure obligations (Carlson 2017) or to refuse to take part in independent testing of algorithm performance. Public bodies are effectively sacrificing their ability to exercise meaningful oversight of algorithm operation and functioning as well as their ability to comply with their own mandated obligations of transparency and reason‐giving.

Algorithm setup often remains undisclosed to the purchasing public body, to those adversely affected by it, and/or to citizens at large. For instance, the methodology behind the workings of the much debated Correctional Offender Management Profiling for Alternative Sanctions (COMPAS) algorithm used in criminal justice in the United States was not disclosed in the face of “machine bias” allegations, despite it being relied upon by courts in sentencing decisions (Carlson 2017; Harvard Law Review 2017). The patterns displayed by COMPAS only became apparent through the matching and comparing of specific predicted recidivism scores with actual recidivism by *ProPublica* journalists a couple of years later, rather than through the direct investigation of the algorithm's source code. While the company published a rebuttal to the findings (Dieterich et al. 2016), where they claim for the relevance of a different standard of fairness (Corbett‐Davies et al. 2016; Dressel and Farid 2018), and with the debate still unresolved, the COMPAS source code continues to be hidden from public view.

While model transparency is in and of itself unlikely to resolve all informational problems pertaining to AI algorithm use, a reliance on proprietary models in the public sector engenders and exacerbates a heavy dependence on private providers truthfully reporting on their models’ functioning (and malfunctioning), highly problematic given the considerable financial and reputational costs at stake shaping disclosure incentives. Without model transparency, independent third parties will not be able to independently audit algorithm functioning (for instance, by testing algorithm operation and predictions on different data) and/or will be left guessing key features when attempting to reverse‐engineer algorithm functioning, while public sector bodies will be left unable to comply with their administrative disclosure duties towards affected citizens.

#### Algorithmic Complexity

Moreover, beyond system feature opaqueness and/or public disclosure issues, there are significant information challenges stemming from ML *model complexity*. Given their architectural complexity and the sheer size of their parameter space, as noted above, it can become next to impossible for human decision‐makers to grasp the intricacy of feature interactions, even in the best case (and unlikely) scenario where the model features do lend themselves to human comprehension and the system's source code is public. In other words, when “hundreds or thousands of features significantly contribute to a prediction, it is not reasonable to expect any user to comprehend why the prediction was made, even if individual weights can be inspected” (Ribeiro, Singh, and Guestrin 2016, 1137). The traditional informational asymmetry problems characteristic of any supervisory system become compounded by difficulties of human information‐processing limitations.

Such limitations become particularly—but not exclusively—emphasized in the case of non‐technical audiences. It is important to remember the context of much algorithm use: while developed by technical experts, these models are sold on to public sector bodies to be used by domain experts. Significant technical expertise asymmetries run to the detriment of such users, further compounded in the public sector by resource shortages and cut‐back pressures on public services, often driving the adoption of algorithms in the public sector. Given model complexity, it is highly unlikely that such users would have the (institutional) capacity to assess these algorithmic outputs and the validity of the parameters on which a prediction was reached. For instance, it is unlikely that when faced with algorithmic risk scores judges will be able to assess the validity of the underlying assumptions, data structure, and key feature interactions of the model producing the scores. Yet, these elements are crucial to the validity of said scores and whether value should be placed on them by human decision‐makers.

Unless models are rendered interpretable and comprehensible by system designers to facilitate user understanding, their inscrutability will invariably entail that such scores are taken at face value. This effectively amounts to introducing pieces of evidence into public sector decision‐making (be it the justice process or other) that cannot be scrutinized and therefore corrected because the parameters on which they were reached are effectively not known/open to user assessment. This can conceivably lead to undue faith among users in algorithmic models and their performance, which is especially problematic given the recurrent and widely reported model failings noted above.

### Debate and Justification: Difficulties in “Interrogating” Algorithmic Decision‐Making

These informational deficits—stemming from algorithms’ inherent opacity, complexity, or proprietary nature—have direct implications for the next phase of accountability: *explanation* or *justification*. The ability to explain the *rationale* leading to a particular decisional outcome is key to interrogating and challenging such outcomes. It is this phase that differentiates accountability from transparency or the mere provision of information above: being able to screen, prod, and pry the *logic* behind actions and decisions.

#### Explainable and Justifiable Algorithms?

Yet explanation is precisely what is especially difficult to achieve due to AI algorithms’ inherent operation. This is especially true for deep learning algorithms. These technical challenges are no doubt also due to the fact that traditionally, the main evaluative criteria for algorithm performance have been based on designer‐determined metrics such as “predictive accuracy” rather than model interpretability. However, a growing realization of the need for interpretability—the need to “provide qualitative understanding between the input variables and the response” (Ribeiro, Singh, and Guestrin 2016, 1136)—is emerging among computer science scholars: “Understanding the reasons behind predictions (…) is fundamental if one plans to take action based on a prediction, or when choosing whether to deploy a new model” (Ribeiro, Singh, and Guestrin 2016, 1135). Developers are increasingly coming to the realization that the continued reliance on—and proliferation of—such systems will come down to user trust in their outputs, with algorithm understandability as an important ingredient thereof. Concerns regarding algorithmic understandability are becoming the focus of a mounting body of work in computer science aimed at developing “explainable AI” approaches (see Guidotti et al. 2019 for an overview), as well as important work demonstrating the value of relying on interpretable algorithms over black‐box ones (Rudin 2019).

As deep learning black‐box models continue to be the most successful algorithmic approach, computer science scholars have been working towards rendering such models more understandable. Ongoing potential approaches range from feature visualization techniques—aimed at visualizing the workings of hidden layers of neuron interactions to render internal processes more explicit (e.g., Zeiler and Fergus 2014 or activation atlases by Google and OpenAI)—to interfacing “black‐box” algorithms with “explainer algorithms” (Ribeiro, Singh, and Guestrin 2016; DARPA XAI program), i.e., essentially using algorithms to explain algorithms. “Explainer algorithms” could conceivably unpack a black‐box for instance, by producing a simpler algorithm (say, a decision tree) to explain that black‐box. The explainer algorithm effectively re‐digests the black‐box features into features that are understandable for the human mind.

Explanation models also have important shortcomings, however. This type of technique may require the “‘input variables’ in the explanations to be different than the features” of the actual black‐box model so as to render them intelligible (Ribeiro, Singh, and Guestrin 2016, 1137). Ex post explanations can rely on different key variables to simulate black‐box decisional logic—in other words, a different model altogether. Inherently therefore, explanation models are not fully faithful representations of the original model, but are rather “approximations” thereof, which necessarily simultaneously reduces their explanatory potential (Rudin 2019). What is more, there are currently no common standards for what is required in terms of an explanation and “no work that seriously addresses the problem of quantifying the grade of comprehensibility of an explanation for humans, although it is of fundamental importance” (Guidotti et al. 2019, 36).

This has led some prominent computer science scholars to question the value of ML *explanation* techniques, emphasizing that the path out of the current conundrum is not to attempt to explain black‐box algorithms but rather when it comes to high‐stakes decision‐making to adopt *interpretable* algorithms—sparser models where it is straightforward to see how different variables are jointly related to each other: “Explanations are often not reliable, and can be misleading (…) If we instead use models that are inherently interpretable, they provide their own explanations, which are faithful to what the model actually computes” (Rudin 2019, 206). Given the serious shortcomings of explanation models (including counter‐factual explanations often presented as a solution for algorithmic accountability), such work is geared at demonstrating that *interpretable* algorithms can be powerful, and do away with the need to rely on overly complex black‐boxes for high‐stakes decision‐making (Dressel and Farid 2018; Rudin 2019). For instance, this work has demonstrated that simple interpretable models (with two and respectively three features) can perform as well as the widely used criminal justice COMPAS algorithm, which instead draws on 137 features.

For the time being however, “[d]espite widespread adoption, machine learning models remain mostly black boxes” (Ribeiro, Singh, and Guestrin 2016, 1135). The discussion above suggests a glaring deficit in algorithm oversight: with black‐box algorithms widely used in the public sector and given that such algorithms are not interpretable, domain experts and other public sector users are unlikely to be able to spot biases and unintended consequences of algorithmic performance in the real world. Regulatory efforts too, have fallen behind practice, and in most jurisdictions there is no legal obligation on the industry to rely on interpretable models, or even to explain their algorithms as a pre‐requisite to commercializing them for public use, even for simpler models. Many algorithms involved in high‐stakes public decision‐making (e.g., public benefits eligibility) routinely lack for instance, audit trails and meaningful explanations of their outcomes (Calo and Citron 2020; Citron 2008).

GDPR, the EU‐wide data protection legislation, does introduce a series of remedies and safeguards on algorithmic decision‐making. Yet these protections—and it is much debated whether these amount to a meaningful right of explanation (Edwards and Veale 2017; Wachter, Mittelstadt, and Floridi 2017)—are restricted to solely automated decisions. In most cases however as noted, high‐stakes algorithmic decisions are not fully automated but rather algorithmic outputs serve as decisional aides, falling outside the scope of these protections. What is more, even when applicable, the legal requirements are ambiguous as to what is required (i.e., “meaningful information about the logic involved”) and risk compromising the value that can be derived from this information: “Because the definition of what constitutes a viable explanation is unclear, even strong regulations such as “right to explanation” can be undermined with less‐than‐satisfactory explanation” (Rudin 2019, 214). In fact, it remains entirely unclear whether what is required is merely a general explanation of system functionality; a post‐hoc explanation model; or, as we have argued here more broadly, whether interpretable models should be given preference to ensure meaningful understanding, from an account‐holding perspective, of algorithmic decision‐making.

#### From Implicit to Explicit Value Trade‐Offs

Model explanation or justification necessarily also extends to an oft‐forgotten aspect of model functioning: value trade‐offs inherent in model design. While AI algorithms are often presented as “neutral devices,” algorithmic systems necessarily encode important value trade‐offs (e.g., recall versus accuracy, precision versus fairness) or even trade‐offs among different notions of the same value (e.g., different notions of fairness). Deciding how to strike the balance among these is necessarily a *political* and not a purely technical act: depending on the value or notion prioritized, the algorithm will reach different results and impose costs and benefits on different individuals or societal groups (see also Bovens and Zouridis 2002; Young, Bullock, and Lecy 2019).

Algorithmic outputs are thus to a significant extent the product of value choices designed within them. In the age of automation, system designers effectively become policy‐makers: “their choices can affect the practical implementation of policy” (Bovens and Zouridis 2002, 181). Theirs is essentially an exercise in so‐called “digital discretion” (Busch and Henriksen 2018) or more specifically in our case, of “artificial discretion” (Young, Bullock, and Lecy 2019). To the extent that, through their choices, system designers promote unintended values and make unsanctioned trade‐offs, this can cause “a shift in public values outside the control of public managers and public policy‐makers” (Busch and Henriksen 2018, 19). It is therefore crucial that such choices are considered, understood, and scrutinized. Doing so is no easy feat: in the case of AI algorithms, such trade‐offs, *implicit* in system design, are obscured by complex layers of code and mathematical computations rendering these decisions inscrutable to outsiders, unless rendered explicit by system engineers. A pre‐requisite to external interrogation of such decisions is being aware of such value choices in the first place.

The relevance of value trade‐off considerations is especially well illustrated by—but certainly not restricted to—much‐debated “machine bias” and fairness aspects. While important technical work is ongoing in computer science to develop technical fixes to issues of “machine bias” and fairness, it is important to acknowledge that “fairness” is not purely a technical, computational solution. While models can produce biased results for technical reasons (inadequate data or sample size—and flowing from that issues with representativeness of models based on such data, poor model fit, etc.), what represents a fair outcome is a contextual and political question. Seemingly technical choices involved in model setup can have important value implications. For instance, technical solutions to avoid gender bias in a model range, among others, from setting up algorithms to provide for parity in outcomes (e.g., that a CV screening algorithm outputs an equal number of qualified male and female candidates), to keeping the system ignorant of the “protected quality” (e.g., not providing it with gender information and omitting correlated variables) so that it cannot use it as a predictive (discriminant) feature. Each of these technical choices will result in different substantive outcomes, and can still produce unfair or discriminatory outcomes, depending on context. For instance, ignoring the “protected quality” can actually result in disadvantaging and discriminating against the respective protected group in specific contexts, as can enforcing outcome parity in some circumstances.

Much of the recent debate surrounding “machine bias” in algorithmic scores has come down to the “tension between competing notions of what it means for a probabilistic classification to be fair to different groups” (Kleinberg, Mullainathan, and Raghavan 2016). In other words, they have come down to *competing value notions*, in this case competing notions of fairness. The COMPAS debate is such an example, where different definitions of fairness are being advanced by different actors to dispute or to defend the validity of algorithmic outputs (Corbett‐Davies et al. 2016; Dressel and Farid 2018). This is no small issue: what is effectively at stake is what standards of fairness should inform a predictive system used in criminal justice—with discussions on this being waged ex post between computer scientists rather than a matter of a priori domain deliberation and interrogation. What is more, computer science work in this context is finding that the evaluative notions involved in these debates entail inherent trade‐offs that cannot be simultaneously met by a model. Key notions of fairness that have been central to these algorithmic debates are essentially incompatible: “it's actually impossible for a risk score to satisfy both fairness criteria at the same time” (Corbett‐Davies et al. 2016; see also Kleinberg, Mullainathan, and Raghavan 2016). This necessarily again highlights that prior deliberation—of the standards that are to be prioritized and inform specific models in different sectors—are crucial steps to adequate model design.

Such choices, implicitly part and parcel of model setup, are shadowed from domain experts, decision‐makers, and regulators. For meaningful algorithmic accountability, these choices need to be *explicitly and transparently spelled out* by system designers, and require of them—but also equally of regulators and political overseers—to remain alert to these aspects and for the discussion and deliberation of these aspects too, when scrutinizing model design and operation.

#### Algorithmic Outputs: What Are the Behavioral Effects on Human Decision‐Making?

Importantly, challenges of *explanation* of algorithmic decision‐making intervene not only with respect to algorithmic models’ setup and operation but also at the *interface* between algorithmic inputs and human decision‐making. As noted above, algorithm results often serve as inputs to human decision‐making. The implication of this is that if we want to understand and explain algorithm‐informed decisions, we need to understand not only AI algorithm operation but also the influence that algorithm results have on (constraining) human decision‐making.

Yet, we know surprisingly little about their effects on decision‐makers and actual decision‐making processes. How, and in which ways, are decision‐makers impacted by algorithmic inputs and recommendations? To what extent do algorithmic results constrain our decision‐making autonomy and/or bias our ability to interrogate them? When judges make decisions using algorithmic risk scores as a source of input into their decision‐making, what influence do these scores have on their decisions?

While the introduction of algorithmic assessments is often justified as a way to render human decisions more objective, it is plausible that algorithms could induce human behavioral biases, with some disconcerting clues in this regard. On the one hand, the reliance on algorithmic outputs could conceivably lead to unjustified deference of the decision‐maker to algorithm predictions: “Automation bias” (Cummings 2006; Lyell and Coiera 2017; Skitka, Mosier, and Burdick 1999) is a well‐documented phenomenon reported in psychological studies manifest in human over‐reliance on automated systems’ recommendations. Humans that follow the automated system slavishly such as a navigation system off a cliff, or pilot error resulting from pilots blindly following navigation systems are such examples. What is more, research into the role of automation suggests that automated tools can potentially serve as “moral buffers” with humans relinquishing responsibility over the decision (Cummings 2006). AI algorithmic systems can then plausibly come with the risk that they suspend our criticism and thereby, not only, as discussed above, our *ability*, but also importantly, our *propensity to interrogate* their outputs. This has strong implications for accountability as it directly pertains to the allocation of decision‐making authority, the locus of decision‐making, and the assignment of responsibility.

Early empirical evidence does seem to suggest that far from being used as “neutral” devices, algorithmic outputs can accentuate disparate and discriminatory outcomes in human decision‐making (Green and Chen 2019). In their experimental study (with lay participants) about receptiveness to algorithmic advice in the context of criminal justice, the authors find evidence of “disparate interactions” with the risk assessment algorithm, leading to higher risk predictions for black defendants and lower risk predictions for white defendants. They document a propensity for selective adherence to the algorithm by participants, resulting in discriminatory outcomes. What is more, the authors also report important limitations in participants’ ability to evaluate the algorithm's performance (97).

These clues are especially worrisome from an accountability perspective as “keeping humans in‐the‐loop” (human intervention) is generally prescribed as a check on automated decision‐making. For instance, the GDPR provides for a right “not to be subject to a decision based solely on automated processing”—with some exceptions (e.g., explicit consent)—as well as requiring the introduction of minimum safeguards in the specified circumstances where fully automated decision‐making is permitted. One such safeguard is “the right to obtain human intervention.” The implicit assumption behind the introduction of these guardrails seems to be that the risk with algorithmic decision‐making lies primarily with fully automated systems—when there is no human decision‐maker in‐the‐loop, and that human intervention is part of the solution. Yet, if it is part of the solution, we lack robust studies as to what—explicit and implicit—impact algorithms actually have on human decision‐makers. This is an area where behavioral public administration scholars could and should play an important role in the future.

### Consequences for Algorithmic Decision‐Making

Finally, the last phase of the accountability process is that of passing judgement—of approving, denouncing, or condemning a particular behavior—and to the extent that the judgement is negative, meaningful accountability dictates the imposition of sanctions and affording redress to those negatively affected.

This dimension of accountability too becomes problematic in the case of algorithmic decision‐making as its operation is closely dependent on the presence of the previous two elements: information and justification. Given compounded informational difficulties, coupled with the considerable explanation problems identified above, this brings related challenges to diagnosing system malfunctioning and (mis)behavior for effective remedial action. A first step to affording redress is diagnosing failure, yet this is no easy feat with algorithmic performance.

Without the ability to understand algorithm features and operation, lay audiences, including individuals adversely affected by algorithmic decisions, cannot meaningfully contest or challenge the decisions taken on their basis and obtain redress. And without these “fire alarms,” algorithmic systems will further lack much‐needed correcting and learning feedback. Without model transparency and explanation of underlying logics of operation, domain experts (public sector users), too, will not be able to “catch” mis‐performing models, leading to undue reliance thereon. This will severely limit their ability to act as meaningful decisional mediators. Under these circumstances having a human putatively in the decisional loop risks becoming a hollow check, where effectively the human mediator has little insight into the system's functioning (or malfunctioning), depriving it of meaningful control.

Importantly however, this does not absolve the public administrator of responsibility—to the contrary. The duty to give reasons for administrative decisions is a hallmark of bureaucratic legitimacy and one that administrators cannot outsource or renege on. The adoption of AI tools within the public sector places responsibility squarely on public sector employees involved in the decision‐making process: *Adopters (managers)* should be held responsible to demand and purchase tools that are compatible with the public nature of their roles and allow public administrators to discharge their continued responsibilities to citizens. This responsibility extends to ensuring that such systems are properly and independently vetted, that their functioning is continuously monitored, and that public sector staff are adequately trained to understand the tools they are to rely on. Correspondingly, in mixed systems, *individual decision‐makers* within the administration are responsible for decisional outcomes. It remains incumbent upon them to understand the broad parameters of model functioning and potential failings—if they rely on, or defer to its outputs in their administrative decision‐making—and to be aware of the potential implications of such systems.

This highlights—yet again—the importance of giving preference to transparent and interpretable tools, which facilitate user understanding, steering away from black‐box models in the public sector (in both the technical and proprietary sense). Simultaneously, it also underscores the responsibility of developers, in collaboration with domain experts, to specifically test and interrogate not only technical but also governance implications (broader considerations of fairness, bias, and transparency) at the model testing and validation phases, as well as of “system architects” to develop models for use in the public sector that are understandable and allow domain experts and affected citizens to diagnose and challenge unanticipated failures during use.

Difficulties with rectification and consequences are already well illustrated by the challenges faced by those adversely affected in being able to contest algorithm‐informed decisions, and to secure redress. To illustrate, despite ongoing controversy and the algorithm's proprietary nature, the COMPAS algorithm continues to be used by US courts. Recently, a federal supreme court (the Wisconsin Supreme Court) held that a trial court's use of the algorithm in sentencing did not breach the defendant's due process rights. The defendant's due process rights were said to be upheld even though the risk assessment score was explicitly referenced by the trial court judge during sentencing yet “the methodology used to produce the assessment *was disclosed neither to the court nor to the defendant*,” being withheld under trade secret protections (Harvard Law Review 2017, 1530). Incidentally, the sentence amounted to 6 years in prison and 5 years of extended supervision for the defendant.

This example sharply highlights the fundamental issues at stake with respect to the ability to challenge the validity of predictive algorithmic outputs, in the absence of information and explanation of how these were derived. Without the “opportunity to understand the basis upon which assessments are made” it becomes “practically impossible for any individual to assert a claim of right, to protest that ‘she is not like that’, or that while she might have been like that in the past, she does not propose to be ‘like that’ in the future” (Yeung 2018, 515). This cuts to the core of individual ability to obtain redress and rectification of wrongful action.

## Conclusion: AI—Accountable Intelligence?

We have seen that challenges arising from algorithm use give rise to deficits that strike at the heart of accountability processes: compounded *informational* problems, the absence of adequate *explanation* or *justification* of algorithm functioning, and ensuing difficulties with diagnosing failure and securing *redress*. At its core, accountability is about answerability—yet current AI algorithm use comes with serious challenges to our collective ability to interrogate (and challenge) algorithmic outcomes.

Our discussion reveals that in the case of algorithms too, like in traditional settings, transparency is a highly necessary but *in*sufficient condition for accountability. In and of itself, transparency of model design in the case of complex AI models—whose features are often opaque and escape interpretability —will fall short by way of providing adequate understanding of algorithmic decision‐making. Model transparency additionally necessitates concerted efforts of “system architects” to explain their models, of the computer science community more broadly to develop models that are understandable and interpretable in the first place, of the industry to systematically adopt such practices, and of public sector purchasers and regulators to demand them. It will also entail critical and ongoing cooperation between system designers and domain experts, and one that spans from the early stages of system design to real‐world implementation (production) and monitoring of system functioning.

Public administration scholars, too, are not exempt from playing an important role. Issues of AI transparency, bias, fairness, and accountability are not purely technical (machine learning) questions but require serious engagement from our discipline and a broader public administration lens (see more broadly, Shark and Shropshire 2019). Accountability challenges raised by the adoption of AI tools within government are inextricably linked to broader questions of bureaucratic legitimacy. AI tools stand to profoundly impact administrative decision‐making and bureaucratic discretion, rendering such developments and their implications fundamental to public administration. The suitability of different AI tools varies across different policy areas and tasks, and the trade‐offs involved need to be well understood and anticipated in order to guide the appropriate diffusion of AI in government (see Young, Bullock, and Lecy 2019 for such a framework as to the utility of “artificial discretion”).

In this connection, the adoption of mixed algorithmic systems that putatively keep a human‐in‐the‐loop, while by virtue of their characteristics (proprietary, non‐transparent, and/or non‐interpretable) severely limit human ability to act as meaningful overseers, is directly problematic. Such transformations are taking place insidiously rather than in a deliberate and considered manner, with bureaucratic actors losing the ability to understand, scrutinize, and exercise meaningful control. These concerns are compounded by the fact that these developments increasingly impact non‐routine, high‐stakes areas (such as criminal justice) where the exercise of human discretion and expertise has been regarded as critical to their implementation. The twin foundations of bureaucratic legitimacy—bureaucratic expertise and accountability—are being simultaneously diminished. As reliance on AI by administrative actors becomes increasingly ubiquitous, remedying these limitations and empowering human decision‐makers to act as meaningful checks will strike at the (continued) legitimacy of the administrative state and its very claim to authority (see also Calo and Citron 2020).

Regulatory efforts are thus vitally needed to ensure that AI tools are brought to bear in a thoughtful and effective manner. The wide adoption of a variety of regulatory tools, for the most part currently lacking, and being proposed by academic and some industry actors, such as: model certification, third‐party independent ongoing testing of model performance, the use of models that provide audit trails of their decision‐making, algorithmic impact assessments pre‐public sector deployment, etc. would further help to bolster algorithmic accountability. Ultimately however, such safeguards alone fail to address the key issue at stake: public sector use of AI tools—where the stakes can be the likes of liberty deprivation, use of force, and welfare or healthcare denial—requires explanation of the *rationale* of individual decision‐making.

The discussion above calls into serious question whether the use of black‐box models is justified in the public sector, especially when interpretable alternatives are available. It seems necessary that in areas where decisions have high‐stakes (individual‐level) implications, algorithms can neither be secret (proprietary) nor uninterpretable. For algorithmic models to be deployed in such contexts requires that models are a priori well understood, that their predictive features are understandable (importantly also to users and domain experts), that value trade‐offs inherent in system design are rendered transparent and adequately deliberated, and that models are validated and audited, in cooperation with domain experts, not only with respect to their strictly technical operation but also with respect to their governance implications.

While regulation is not necessarily a popular topic when it comes to technological innovation, it is important to remember what is at stake. When black‐box algorithmic systems get assimilated into the public sector, they permeate the very fabric of our institutions. They permeate our justice system, our education, our law enforcement, all the while remaining inscrutable to challenge. And therein lies the rub.

## Funding

This article is part of a project that has received funding from the European Research Council (ERC) under the European Union's Horizon 2020 research and innovation programme (grant agreement 716439).
